# Comprehensive landscape and future perspectives of circular RNAs in colorectal cancer

**DOI:** 10.1186/s12943-021-01318-6

**Published:** 2021-02-03

**Authors:** Fei Long, Zhi Lin, Liang Li, Min Ma, Zhixing Lu, Liang Jing, Xiaorong Li, Changwei Lin

**Affiliations:** 1 Department of Gastrointestinal Surgery, The Third Xiangya Hospital of Central South University, Changsha, Hunan 410013 P.R. China; 2 Department of Gastrointestinal Surgery, The First Affiliated Hospital of The University of South China, Hengyang, Hunan 421001 P.R. China; 3 Department of Pediatrics, Xiangya Hospital, Central South University, Changsha, Hunan 410008 P.R. China; 4 Class 25 Grade 2016, The Five-Year Program in Clinical Medicine, School of Medicine, University of South China, Hengyang, Hunan 421001 P.R. China; 5 School of Life Sciences, Central South University, Changsha, 410078 Hunan China

**Keywords:** circRNAs, ceRNAs, Colorectal cancer, Dysregulation, Functions, Mechanisms, Perspective

## Abstract

Colorectal cancer (CRC) is a common hereditary tumor that is often fatal. Its pathogenesis involves multiple genes, including circular RNAs (circRNAs). Notably, circRNAs constitute a new class of noncoding RNAs (ncRNAs) with a covalently closed loop structure and have been characterized as stable, conserved molecules that are abundantly expressed in tissue/development-specific patterns in eukaryotes. Based on accumulating evidence, circRNAs are aberrantly expressed in CRC tissues, cells, exosomes, and blood from patients with CRC. Moreover, numerous circRNAs have been identified as either oncogenes or tumor suppressors that mediate tumorigenesis, metastasis and chemoradiation resistance in CRC. Although the regulatory mechanisms of circRNA biogenesis and functions remain fairly elusive, interesting results have been obtained in studies investigating CRC. In particular, the expression of circRNAs in CRC is comprehensively modulated by multiple factors, such as splicing factors, transcription factors, specific enzymes and cis-acting elements. More importantly, circRNAs exert pivotal effects on CRC through various mechanisms, including acting as miRNA sponges or decoys, interacting with RNA binding proteins, and even translating functional peptides. Finally, circRNAs may serve as promising diagnostic and prognostic biomarkers and potential therapeutic targets in the clinical practice of CRC. In this review, we discuss the dysregulation, functions and clinical significance of circRNAs in CRC and further discuss the molecular mechanisms by which circRNAs exert their functions and how their expression is regulated. Based on this review, we hope to reveal the functions of circRNAs in the initiation and progression of cancer and highlight the future perspectives on strategies targeting circRNAs in cancer research.

**Supplementary Information:**

The online version contains supplementary material available at 10.1186/s12943-021-01318-6.

## Background

Currently, colorectal cancer (CRC) is the third most common malignancy and the second leading cause of cancer-related death worldwide [1]. Moreover, the incidence and mortality rates of CRC are still rapidly increasing in many developing countries, and the public health burden of CRC is expected to increase by 60% in 2030, with more than 2.2 million new cases diagnosed and 1.1 million deaths [2]. Following the implementation of early screening and progress in standardized treatments, the prognosis of patients with CRC has significantly improved, with 5-year survival rates of early-stage patients approaching 90% [3]. However, most patients with CRC are diagnosed at advanced stages due to the lack of distinctively incipient symptoms and the limitations of early detection and screening. The prognosis of these patients is still poor, particularly in patients with late-stage disease and distant metastasis, whose 5-year survival rate is only 13.1% [3]. To further prolong the survival time and improve the quality of life of patients with CRC, studies aiming to elucidate the molecular mechanisms underlying the occurrence and development of CRC and to identify new biomarkers for the early diagnosis and prediction and novel targets for the effective prevention and treatment of CRC recurrence and metastasis are important and urgently needed.

**Fig. 1:**
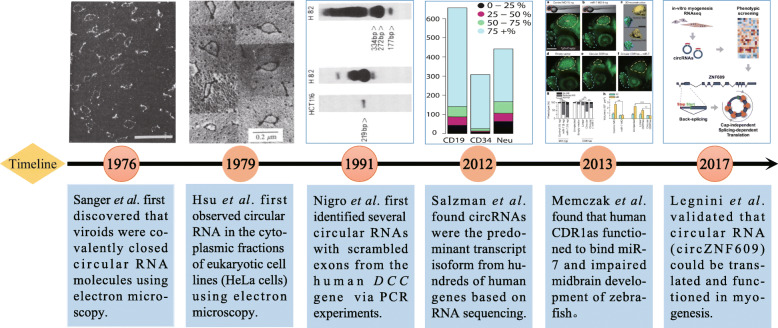
History of the discovery and development of circRNAs. | Representative milestone events leading to the discovery and development of circRNAs are enumerated in the figure

Circular RNAs (circRNAs) objectively exist in organisms (Fig. 1). As early as 1976, Sanger et al. first discovered single-stranded circRNA molecules in viroids, although they are not produced by a backsplicing mechanism [4]. Initially, circRNAs were mainly considered ‘byproducts’ or ‘junk’ generated by abnormal splicing events, and only a rare few circRNAs (e.g., circSRY, circDCC and circEST1) were thought to have possible functions [5, 6]. In recent years, high-throughput RNA sequencing (RNA-seq), circRNA-specific microarrays and bioinformatics analyses have identified thousands of circRNAs in eukaryotes such as fungi, protists, plants, worms, insects, fish and mammals [7–9] and revealed their cell-, tissue-, and timing-specific expression patterns [10, 11]. Moreover, numerous studies have confirmed that circRNAs perform regulatory functions in tissue development (e.g., neurogenesis and myogenesis) [12–15], organism aging [16, 17], and the incidence of diseases, such as tumors, neurological disorders, diabetes mellitus, cardiovascular diseases and chronic inflammatory diseases [18, 19]. Although circRNAs have been less well characterized in other human diseases, most studies have focused on the role of circRNAs in cancers (e.g., CRC, hepatocellular carcinoma, gastric cancer, glioma, etc.) and reported functions in tumorigenesis and metastasis in which individual circRNAs have been identified as either oncogenes or tumor suppressors [20, 21].

Studies investigating circRNAs in CRC rapidly accumulated after 2018. We identified nearly 200 papers by using the terms “circRNAs and colorectal cancer” while searching PubMed and Web of Science, sought out similar articles in PubMed, and checked the cited literature in the references until August 2020. This comprehensive review summarizes recent progress in our understanding of the dysregulation, functions and clinical implications of circRNAs in CRC and discusses the upstream mechanisms of circRNA biogenesis and downstream mechanisms of circRNA functions. In particular, we highlight the future perspectives of circular RNAs in cancer to lay a foundation and identify directions for further research on circRNAs.

## Overview of circRNAs

### Biogenesis and degradation of circRNAs

Most circRNAs are derived from known protein-coding genes with highly active promoters and consist of a single exon or multiple exons [22, 23]. CircRNAs generally have long introns flanking the exons and are mainly generated by backsplicing, which differs from the canonical linear splicing mechanism [24]. During backsplicing, a downstream splice-donor (SD) site is covalently linked to an upstream splice-acceptor (SA) site, resulting in the formation of exonic circRNAs (EcircRNAs) or exon-intron circRNAs (EIcircRNAs). Additionally, circRNAs are produced from lariat precursors when the lariats undergo internal backsplicing [25, 26]. The lariat precursors are usually created by exon-skipping, which is an event during which alternative exons are spliced out of the final mRNA products and contained within the excised lariats during linear splicing [25]. Finally, intronic lariat precursors that escape the debranching step of canonical linear splicing lead yield so-called circular intronic RNAs (CiRNAs) [27]. Although these hypotheses have been widely accepted, the underlying mechanisms of circRNA biogenesis remain fairly elusive.

Following biogenesis, most circRNAs, except for intron-containing circRNAs, are transported from the nucleus to the cytoplasm by the ATP-dependent RNA helicase DDX39A (also known as URH49) and spliceosome RNA helicase DDX39B (also known as UAP56) in a size-dependent manner [28]. CircRNAs exhibit an exceptionally high stability, which is presumably attributed to their covalently closed ring structure, which protects these molecules from exonuclease-mediated degradation [12, 22]. The mechanisms underlying their degradation have only recently begun to be elucidated. Preliminary data show that the RNase P-MRP complex [29], RNase L [30], and the Argonaute 2 (AGO2) protein [31, 32] may participate in the cleavage of specific circRNAs under specific circumstances. For example, circRNAs containing the RNA modification N^6^-methyladenosine (m^6^A) are subject to endoribo-nucleolytic cleavage by the RNase P-MRP1 complex in a manner depending on YTHDF2 and HRSP12 [29]. In addition, some circRNAs (e.g., ciRS-7, ciRS-122 and circHIPK3) are actively exported to the extracellular space in exosomes, which may be a possible mechanism by which cells clear redundant circRNAs [33–37]. More importantly, ciRS-7 and ciRS-122 retain their circular structure in exosomes and function upon release into recipient cells, indicating that exosomal circRNAs may also play a role in intercellular communication [36, 37].

### Classification and functions of circRNAs

According to their biogenesis mechanisms, circRNAs are usually classified into the following three categories: EcircRNAs in which the internal intron is spliced out; EIcircRNAs, namely, circRNAs that contain sequences derived from both exons and introns [38]; and CiRNAs, which contain sequences originating exclusively from introns [27]. EcircRNAs account for most circRNAs and are generally localized to the cytoplasm despite the lack of a cap and poly (A) tail [7, 12, 39]. EcircRNAs mainly function by interacting with microRNAs (miRNAs) or RBPs [12, 23, 24]. In contrast, EIcircRNAs and CiRNAs predominantly reside in the nucleus and possess fewer miRNA binding sites than EcircRNAs [27, 38]. Functionally, EIcircRNAs (e.g., circEIF3J and circPAIP2) promote the transcription of their host genes by interacting with the U1 small nuclear ribonucleoprotein (snRNP) [38], while CiRNAs (e.g., ci-ankrd52) exert a positive regulatory effect on the noncoding transcripts of their parental genes by regulating RNA polymerase II-mediated transcription [27]. Similar to long noncoding RNAs (lncRNAs) [40], circRNAs have also been divided into five types, namely, exonic, intronic, sense overlapping, antisense, and intergenic, based on their position in the genome relative to the protein-coding genes [41]. The proportions of these five types vary from species to cells, and different types perform various functions.

To date, numerous studies have revealed the broad expression of endogenous circRNAs in all human tissues, and circRNAs have increasingly been implicated in the regulation of neuronal functions, cell proliferation and transformation and innate immunity through various molecular mechanisms [42]. For example, hsa_circ_0001946, also known as ciRS-7 or CDR1as, is perhaps the most well-characterized circRNA and has been shown to regulate neuronal development [12, 13, 32, 43], insulin secretion [44, 45] and tumorigenesis [46, 47] by sponging or decoying miR-7 and miR-7-independent mechanisms. Importantly, hsa_circ_0000284 (circHIPK3) promotes insulin secretion and improves β cell function by sponging mir-124-3p and mir-338-3p in individuals with diabetes mellitus [44]; hsa_circ_0000284 also plays dual regulatory roles in the growth and aggressiveness of cancer by sequestering multiple miRNAs [48–51]. However, the proposed biological functions and mechanisms of action of only a minor fraction of the circRNAs identified to date have been determined, and most circRNAs still require further studies.

## CircRNAs and CRC

### Aberrant expression of circRNAs in CRC

RNA-seq and circRNA-specific microarrays are the most commonly used methods for genome-wide profiling of circRNAs, and thousands of circRNAs have been identified in CRC tissues, cells, exosomes, and blood from patients with CRC (Additional file 1) [52, 53]. Subsequently, by comparing the expression levels between cancer and normal groups, hundreds of differentially expressed circRNAs (DECs) have been selected according to the fold change, *p*-value or fluorescence value (Additional file 1).

#### Dysregulated circRNAs in CRC tissues

Tian et al. applied a microarray containing 162,351 human circRNA probes to screen the expression profiles of circRNAs in 4 matched CRC tissues and adjacent normal tissues [54]. Based on the filter criteria of |FC| ≥ 2, *P* < 0.05, and fluorescence intensity ≥100, 13,198 DECs were identified, including 6697 upregulated and 6501 downregulated DECs [54]. Zhou et al. performed RNA-seq to profile mRNA and circRNA expression in 8 pairs of CRC tissues and their adjacent nontumor tissue samples; 31,557 circRNAs were detected in the screening. Of these circRNAs, 81% were derived from protein-coding exons, 5% were derived from intronic sequences, and 14% were derived from other sequences [55]. Consistent with these findings, Tian et al. identified 265 DECs and thousands of lncRNAs and mRNAs using a microarray assay; among the dysregulated circRNAs, 75.85% were exonic circRNAs, 11.32% were intronic circRNAs, 7.92% were sense overlapping circRNAs, 3.4% were antisense circRNAs, and 1.51% were intergenic circRNAs [41]. Based on the RNA-seq data and differential expression analysis of 20 paired frozen tissues, Ju et al. identified 103 abnormally expressed circRNAs (48 upregulated and 55 downregulated) between recurrent and nonrecurrent tumor tissues [56]. In addition, Xu et al. detected 66,855 circRNAs in tissue samples from three patients with CRC and liver metastases and three matched patients with CRC using secondary sequencing; 113 of these circRNAs were DECs, and 92 upregulated and 21 downregulated DECs were identified [57]. Zeng et al. also detected 431 DECs (192 upregulated and 239 downregulated) in CRC tissues from patients with lung metastasis compared with CRC tissues from patients without metastasis using a high-throughput microarray assay [58]. Interestingly, Yuan et al. and Chen et al. searched for tumorigenesis- and metastasis-associated circRNAs in CRC mouse models using RNA-seq; finally, mmu-circ-001226/mmu-circ-000287 and circ-NSD2 (mm9_circ_003195) were identified to be associated with CRC tumorigenesis and metastasis, respectively [59, 60].

#### Dysregulated circRNAs in CRC cells

Jin et al. used high-throughput RNA-seq to analyze circRNA expression profiles in the normal colon epithelial cell line FHC and seven CRC cell lines (LoVo, SW480, HCT116, SW620, HT29, HCT8, and DLD1) and identified 112 DECs between the FHC cell line and CRC cell lines using the following selection criteria: *P* < 0.01 and |Log2FC| ≥1 [61]. Using RNA-seq and a differential expression analysis, Jiang et al. investigated the circRNA profiles in two CRC cell lines (the primary tumor cell line SW480 and its metastatic cell line SW620) and identified a large set of 2919 circRNAs with significantly differential expression patterns compared to normal cells (NCM460) [62]. In addition, the authors identified a set of 623 DECs that differed between the SW480 and SW620 cell lines [62]. In particular, a microarray analysis of circRNA expression profiles in CRC cells (HCT116) identified several DECs associated with 5-fluorouracil (5-FU)-based chemoradiation resistance [63, 64]. More interestingly, Dou et al. observed a significant global downregulation of circRNAs in KRAS mutant colon cancer cells (e.g., DLD-1, DKO-1, and HCT116 cells) compared to KRAS wild-type cells (e.g., DKs-8 and HKe3 cells), indicating a broad effect of mutant KRAS on circRNA abundance [34].

#### Dysregulated circRNAs in blood from patients with CRC

Ye et al. performed a circRNA microarray analysis using 8 plasma samples, including four samples from patients with CRC and four samples from healthy controls, and detected 204 DECs between the CRC and normal plasma samples; among these DECs, 178 were upregulated and 26 were downregulated in plasma from patients with CRC [65]. These circRNAs, including hsa_circ_0082182, hsa_circ_0000370, and hsa_circ_0035445, may play a role in the pathogenesis of CRC and might be potential noninvasive biomarkers for the detection of CRC.

#### Dysregulated circRNAs in CRC exosomes

Exosomes are extracellular vesicles or microvesicles secreted by various cells that display differential expression of exosome markers, such as TSG101, HSP70, CD9, and CD63, but do not express albumin or calnexin [37, 66, 67]. Usually, exosomes are translucent cup-shaped or spherical structures in transmission electron microscopy (TEM) images with diameters ranging from 30 to 150 nm [68, 69]. In recent years, exosomes have been reported to be carriers delivering miRNAs, lncRNAs, proteins and even circRNAs for intercellular signal transduction [68–70]. Dou et al. detected circRNAs in secreted exosomes from three colon cancer cell lines (DLD-1, DKO-1, and DKs-8), and circRNAs were more abundant in extracellular vesicles than in cells [34]. By conducting an RNA-seq analysis of 50 CRC and 50 healthy control serum exosome samples, Xie et al. identified 1924 CRC-related circRNAs [71]. These circRNAs were widely distributed across all chromosomes and included 33.2% intronic circRNAs, 31.6% exonic circRNAs, 18.3% antisense circRNAs, and 16.9% circRNAs from other sources [71]. According to the statistical criteria of |FC| ≥2.0 and *P* ≤ 0.05, 122 DECs, including 100 upregulated and 22 downregulated circRNAs, were selected [71]. Feng et al. also identified 3864 significantly dysregulated exosomal circRNAs in plasma from patients with CRC, of which 1889 circRNAs were upregulated and 1975 circRNAs were downregulated [66]. Interestingly, Hon et al. performed a circRNA microarray using exosomal RNAs from HCT116-resistant and HCT116-parental cells and identified 105 significantly upregulated and 34 downregulated circRNAs in HCT116-resistant exosomes [67]. These circRNAs might be potential liquid biopsy indicators for the diagnosis of CRC and may play regulatory roles in the initiation (e.g., circPNN), progression (e.g., circIFT80) or chemoresistance (e.g., circ_0000338) of CRC.

### Biological functions of circRNAs in CRC

**Fig. 2:**
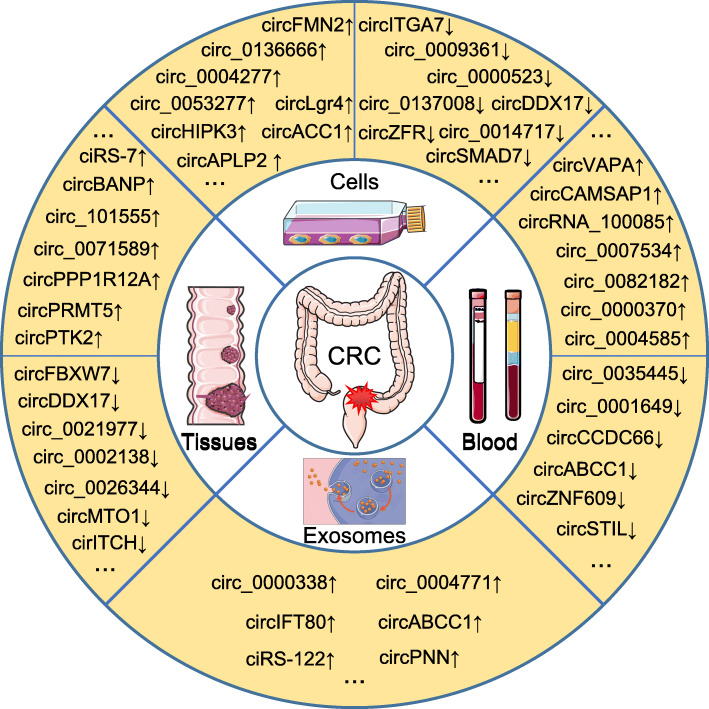
Aberrant expression of circRNAs in colorectal cancer. | CircRNAs are differentially expressed in CRC tissues, cells, exosomes, and blood from patients with CRC compared with normal controls. Representative dysregulated circRNAs are listed in the figure; ‘↑’ indicates upregulated, ‘↓’ indicates downregulated, and ‘...’ represents other dysregulated circRNAs that are not listed

**Fig. 3:**
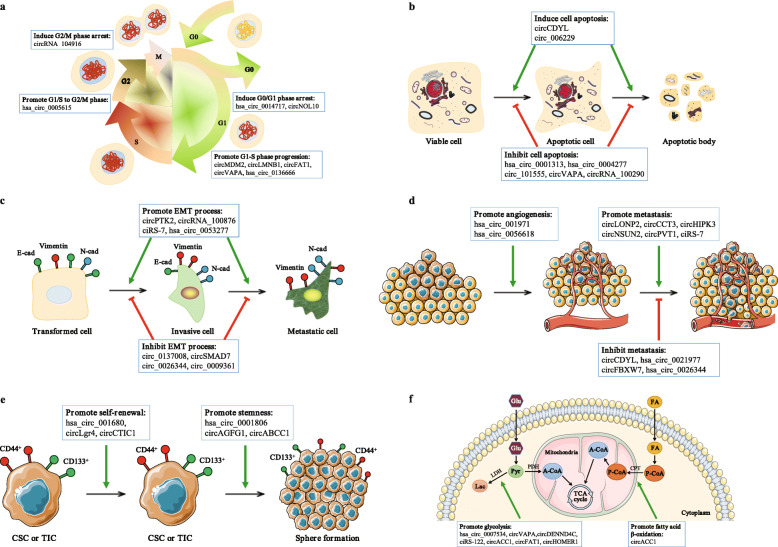
Biological functions of circRNAs in colorectal cancer. **a** | circRNAs regulate the cell cycle by inducing cell cycle arrest (e.g., circNOL10) or promoting cell cycle progression (e.g., circ-MDM2). **b** | circRNAs also regulate cell apoptosis by inducing (e.g., circCDYL) or inhibiting (e.g., circVAPA) cell apoptosis. **c** | Some circRNAs modulate the epithelial-mesenchymal transition (EMT) process by promoting (e.g., circPTK2) or inhibiting (e.g., circSMAD7) EMT; E-cad = E-cadherin, N-cad = N-cadherin. **d** | A few circRNAs have been shown to promote tumor angiogenesis and metastasis (e.g., circ-001971), while other circRNAs inhibit tumor metastasis (e.g., circ-FBXW7). **e** | Several circRNAs promote the self-renewal of colorectal cancer stem cells (CSCs) or tumor-initiating cells (TICs) and maintain the stemness of CRC cells (e.g., circLgr4 and circAGFG1). **f** | Finally, certain circRNAs modulate fatty acid β-oxidation (FAO) or glycolysis in CRC cells, resulting in profound changes in cellular lipid storage and increased cell growth (e.g., circACC1); Glu = glucose, Pyr = pyruvate, Lac = lactate, A-CoA = acetyl-CoA, P-CoA = palmitoyl CoA, FA = fatty acid, LDH = lactate dehydrogenase, PDH = pyruvate dehydrogenase, CPT = carnitine palmitoyltransferase, TCA cycle = tricarboxylic acid cycle

Based on validation experiments such as Northern blot, reverse transcription-quantitative PCR (RT-qPCR), or droplet digital PCR (ddPCR) analyses, many key circRNAs have been confirmed to be significantly dysregulated in CRC tissues, cells, blood, and exosomes (Fig. 2). Among these circRNAs, most circRNAs, including hsa_circ_0014717 [72], circHIPK3 [50], hsa_circ_0009361 [73], etc., exhibit the same expression patten in CRC tissues and cells. Some circRNAs are aberrantly expressed in both tissues and blood from CRC patients as exemplified by circCAMSAP1 [55]. In particular, circVAPA is upregulated in CRC tissues and cells and plasma from patients with CRC [74, 75]; circIFT80 is significantly upregulated in CRC tissues, cell lines, and serum exosomes compared with normal controls [66]. Inexplicably, the level of circABCC1 was increased in CRC tissues and exosomes [76] but decreased in plasma from patients with CRC [77]. More importantly, over 100 hub circRNAs have been shown to participate in the tumorigenesis, metastasis or chemoradiation resistance of CRC based on the results of in vitro and in vivo functional experiments (Fig. 3, Additional file 2).

#### Oncogenic functions

Generally, upregulated circRNAs function as oncogenes in CRC and promote cell proliferation, migration and invasion but inhibit cell cycle arrest and apoptosis (Fig. 3a, b and d; Additional file 2). For instance, hsa_circ_0005075 promotes tumor progression by increasing the proliferation, migration, and invasion of CRC cells [78, 79]; the knockdown of hsa_circ_0000284 (circHIPK3) markedly suppresses CRC cell proliferation, migration, and invasion, induces apoptosis in vitro and inhibits CRC growth and metastasis in vivo [50, 51]. Moreover, some circRNAs modulate fatty acid β-oxidation or glycolysis in CRC cells, resulting in profound changes in cellular lipid storage and increased cell growth [75, 80–84] (Fig. 3f, Additional file 2). These functions are exemplified by hsa_circ_0000759 (circACC1), which is a circular RNA implicated in lipid metabolism that promotes glycolysis and fatty acid oxidation in HCT116 cells [80]. Consistent with the observed metabolic changes, the silencing or forced expression of circACC1 results in the inhibition and promotion of CRC growth, respectively [80]. Furthermore, several circRNAs promote the self-renewal of colorectal cancer stem cells (CSCs) [85] or tumor-initiating cells (TICs) [86] and contribute to cell stemness, sphere formation, and metastasis [76] (Fig. 3e; Additional file 2). For example, hsa_circ_02276 (circLgr4) was identified as a circRNA expressed at high levels in colorectal tumors and colorectal CSCs; circLgr4 knockdown inhibits colorectal CSC self-renewal, colorectal tumorigenesis and invasion, while circLgr4 overexpression exerted the opposite effects [85]. Finally, certain circRNAs promote angiogenesis in CRC, which is a key event maintaining tumor cell survival and aggressiveness [87, 88] (Fig. 3d; Additional file 2). According to Chen et al., circ-001971 relieved the miR-29c-3p-induced inhibition of VEGFA, which is among the most important tumor cell-secreted proangiogenic factors, thereby increasing the proliferation, invasion and angiogenesis of CRC [88].

#### Tumor-suppressor function

Downregulated circRNAs typically function as tumor suppressors in CRC that suppress cell proliferation, migration and invasion while inducing cell cycle arrest and apoptosis (Fig. 3a, b and d; Additional file 2). For example, ectopic hsa_circ_0026782 (circITGA7) expression inhibits the growth and metastasis of CRC cells in vitro and in vivo, whereas the knockdown of circITGA7 promotes the proliferation and migration of CRC cells in vitro and increases CRC growth in vivo [89, 90]. Moreover, hsa_circ_0008285 (circCDYL) decreases cell viability, impairs migration and invasion behaviors, and promotes cell apoptosis in CRC [91, 92]; hsa_circ_0026344 overexpression decreases the growth and invasion of CRC cells while inducing cell apoptosis in vitro and inhibiting CRC growth in vivo [93, 94]. In addition, some circRNAs inhibit the epithelial-mesenchymal transition (EMT), which is a process strongly correlated with metastasis, in CRC cells [73, 94, 95] (Fig. 3c; Additional file 2). Geng et al. confirmed that the overexpression of hsa_circ_0009361 suppresses the proliferation, EMT, migration, and invasion of CRC cells by upregulating the expression of APC2 and inhibiting the activity of the Wnt/β-catenin pathway, which is capable of inducing EMT [73].

Notably, Wu et al. observed the upregulation of hsa_circ_0000615 (circZNF609), a circRNA with multiple functions in human diseases, in CRC tissues, and the knockdown of circZNF609 inhibited the migration of HCT116 cells by suppressing Gli1 expression via microRNA-150 [96]. In contrast, Zhang et al. verified that circZNF609 was significantly downregulated in CRC tissues, cells and serum from patients with CRC compared with normal controls; the overexpression of circZNF609 inhibits cell proliferation and induces apoptosis by upregulating the expression of the pro-apoptotic protein Bax, downregulating the expression of the anti-apoptotic protein Bcl-2, and upregulating p53 [97]. Therefore, the expression and role of circZNF609 in CRC require further investigation.

#### Modulating chemoresistance

The standard treatment for advanced CRC is surgery combined with chemotherapy (pre- or postoperation) in which 5-FU and platinum (e.g., oxaliplatin) are the first-line drugs [98, 99]. However, chemoresistance has substantially affected the effectiveness of CRC treatments, resulting in tumor relapse accompanied by metastasis. Based on accumulating evidence, circRNAs may function as essential regulators of drug resistance in cancers, including CRC [37, 100–105] (Additional file 2). For example, Lai et al. observed the increased expression of hsa_circ_0079662 in oxaliplatin-resistant CRC cell lines, and the knockdown of hsa_circ_0079662 inhibited the proliferation, migration, and invasion of these cells [105]. As shown in a study by Ren et al., hsa_circ_0002211 (circDDX17) overexpression increases 5-FU sensitivity and the apoptosis rate while blocking tumor growth and increasing 5-FU sensitivity in vivo [103]. Moreover, Jian et al. found that hsa_circ_0000598 (circ_001680) increased the colorectal CSC cell population in CRC and induced irinotecan therapeutic resistance by regulating the expression of the miR-340 target gene BMI1 [102]. Furthermore, Wang et al. confirmed that exosomes from oxaliplatin-resistant CRC cells transfer hsa_circ_0005963 (ciRS-122) to oxaliplatin-sensitive cells, thereby enhancing glycolysis and drug resistance by promoting PKM2 expression [37].

#### Regulating radiosensitivity

Radiotherapy is among the most important treatments for CRC in combination with surgery and/or chemotherapy. Unfortunately, CRC radioresistance substantially limits the efficacy of radiotherapy, such as reducing the tumor volume and preventing postoperative recurrence [106, 107]. Based on accumulating evidence, ncRNAs, including miRNAs, lncRNAs, and circRNAs, play increasingly important roles in the process regulating radiation responses [108–110] (Additional file 2). Li et al. observed that after carbon ion irradiation, hsa_circ_0095155 (circRNA CBL.11) expression was increased in CRC cells and suppressed the proliferation of CRC cells through the miR-6778-5p/YWHAE axis [111]. Another example is hsa_circ_0001313 (circCCDC66), which promotes CRC growth and metastasis [112, 113] and induces drug-resistance [37]. Wang et al. further confirmed that circCCDC66 expression was remarkably increased in radioresistant colon cancer tissues compared with radiosensitive tissues and was expressed at high levels in colon cancer cells exposed to radiation [114]. The knockdown of circCCDC66 reduces cell viability and the colony formation rate and increases caspase-3 activity in irradiated colon cancer cells by negatively regulating miR-338-3p, suggesting that circCCDC66 plays a role in radiotherapy resistance [114].

### Molecular mechanisms of circRNAs in CRC

#### Upstream regulator of circRNA biogenesis

**Fig. 4:**
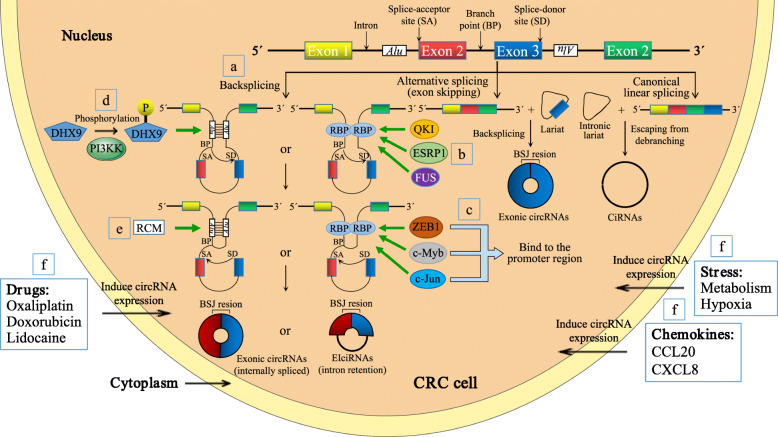
Biogenesis and regulation of circRNAs in colorectal cancer. **a** | During backsplicing (left panel), base pairing between inverted repeat elements (such as *Alu* elements) or the dimerization of trans-acting RNA binding proteins (RBPs) brings a downstream splice-donor site (SD) into close proximity with an upstream splice-acceptor site (SA). Then, an upstream branch point (BP) attacks a downstream SD site, which subsequently attacks an upstream SA site to result in the formation of exonic circRNAs (EcircRNAs) or exon-intron circRNAs (EIcircRNAs). CircRNAs are also generated from a lariat created by an exon-skipping event during linear splicing (middle) or an intronic lariat that escapes from the debranching step of canonical linear splicing (right panel). BSJ = backsplice junction, CiRNA = circular intronic RNA. **b** | RBPs (e.g., QKI, ESRP1 and FUS) promote the production of circRNAs by binding the flanked intron regions of circRNAs. **c** | Transcription factors (e.g., ZEB1, c-Myb and c-Jun) have been shown to increase circRNA expression by physically binding the promoter region of circRNAs. **d** | PI3KK-mediated DHX9 phosphorylation may impair the capacity of DHX9 to resolve RNA pairing, inducing the expression of circCCDC66. **e** | The base pairing between reverse complementary sequences (RCMs), particularly *Alu* elements located in the upstream and downstream introns, bring flanking introns in close proximity to promote backsplicing. **f** | Certain drugs, chemokines and stress induce or inhibit the expression of circRNAs through unknown mechanisms

The mechanism underlying circRNA biogenesis, particularly the process regulating circRNA formation, remains largely unknown. Notably, relevant studies concerning CRC may provide insight into the molecular mechanism of circRNA dysregulation (Fig. 4). RBPs (e.g., QKI and FUS) play essential roles in the production of circRNAs by binding the flanking intronic regions of circRNAs [115, 116] (Fig. 4b). Consistent with these results, Zhou et al. found that the silencing of QKI reduced circCAMSAP1 expression in CRC cells by approximately 20% [55]. More importantly, the authors identified the splicing factor ESRP1, which mediated the biogenesis of circCAMSAP1 in CRC cells by interacting with “GGT-rich” motifs in the flanking intronic regions of circCAMSAP1 [55]. ESRP1 knockdown reduces circCAMSAP1 expression in CRC cells by approximately 50%, and ESRP1 regulates the expression of circBIRC6 in human embryonic stem cells [117]. Consistent with previous results, Han et al. documented that the biogenesis of hsa_circ_0008558 (circLONP2) was modulated by FUS (a well-known proto-oncogene) and that circLONP2 was downregulated upon FUS knockdown with a small interfering RNA (siRNA) [118]. Thus, multiple splicing factors may participate in the biogenesis of individual circRNAs; meanwhile, certain splicing factors may also regulate the expression of different circRNAs in various diseases.

Some transcription factors are also implicated in the biogenesis of specific circRNAs (Fig. 4c). According to Ren et al., hsa_circ_0001178 promotes the metastatic dissemination of CRC by increasing ZEB1 expression and concurrently sponging miR-382/587/616 [119]. Subsequently, ZEB1, a key transcription factor, also increases hsa_circ_0001178 expression by physically binding the promoter region of hsa_circ_0001178 [119]. Therefore, a positive feedback loop is formed between hsa_circ_0001178 and ZEB1 that amplifies the pro-metastatic role of the hsa_circ_0001178/miRNAs/ZEB1 ceRNA axis in CRC. As shown in a study by Li et al., c-Myb increases the expression of circHIPK3 by directly binding its promoter region [50], which is consistent with previous reports [120, 121]. Moreover, Li et al. reported that the expression of circACC1, which plays a critical role in CRC cellular responses to metabolic stress, is preferentially mediated by the transcription factor c-Jun over ACC1 in response to serum deprivation [80].

Another pioneer study showed that ATP-dependent RNA helicase A (also known as DHX9), which unwinds the helical structure of double-stranded RNA (dsRNA), suppresses the biogenesis of circRNAs that rely on base pairing between inverted repeats [122]. Consistent with this finding, Lin et al. proposed that the expression of circCCDC66 is induced by oxaliplatin through PI3KK-mediated DHX9 phosphorylation, which may impair the capacity of DHX9 to resolve RNA pairing [104] (Fig. 4d). Their findings provide a novel and alternative mechanism explaining the link between oncogenic circRNAs and the upregulated expression of DHX9 in cancers.

In addition, base pairing between reverse complementary sequences (RCMs), particularly *Alu* elements located in the upstream and downstream introns, bring flanking introns in close proximity to promote backsplicing [9, 25, 123] (Fig. 4a and e). According to a study by Li et al., the presence of full-length flanking introns led to high circITGA7 expression in CRC cells, and no expression was observed when the flanking introns were deleted [89]. However, the deletion of *Alu* sequences alone had a minimal effect on circITGA7 formation because *Alu* sequences exist only in the upstream intron of circITGA7, not in the downstream intron [89]. Therefore, other RCMs may be located in the flanking introns and facilitate backsplicing, or other factors may mediate the circularization of circITGA7.

Finally, circRNA formation is induced by certain drugs [83, 124, 125], chemokines [94] and stress [80, 126] (Fig. 4f). Chaudhary et al. observed the upregulation of hsa_circ_0027492 (circMDM2) in three p53 wild-type CRC lines (HCT116, RKO and SW48 cells) after treatment with the DNA-damaging agent doxorubicin, and the overexpression of circMDM2 was induced in a p53-dependent manner and was not restricted to CRC cells [125]. Shen et al. observed the downregulation of circ_0026344 following costimulation with CCL20 and CXCL8 (two well-known chemokines), leading to EMT in CRC cells [94]. Shi et al. verified that a hypoxic tumor microenvironment played a vital role in inducing the expression of hsa_circ_0000826 in CRC tissues, which promotes CRC tumorigenesis and liver metastasis in vitro and in vivo [126].

Despite the mechanisms proposed above, the detailed mechanisms underlying circRNA dysregulation are far from understood and require further investigation. Additionally, the disruption of the balance among circRNA biogenesis, intracellular localization, and turnover requires the synergistic effects of multiple factors. Accordingly, a study by Kramer et al. further suggested that the generation of many circRNAs is comprehensively modulated by a combination of cis-acting elements and trans-acting splicing factors, including hnRNPs and SR proteins [127].

#### Downstream mechanisms of circRNA functions

**Fig. 5:**
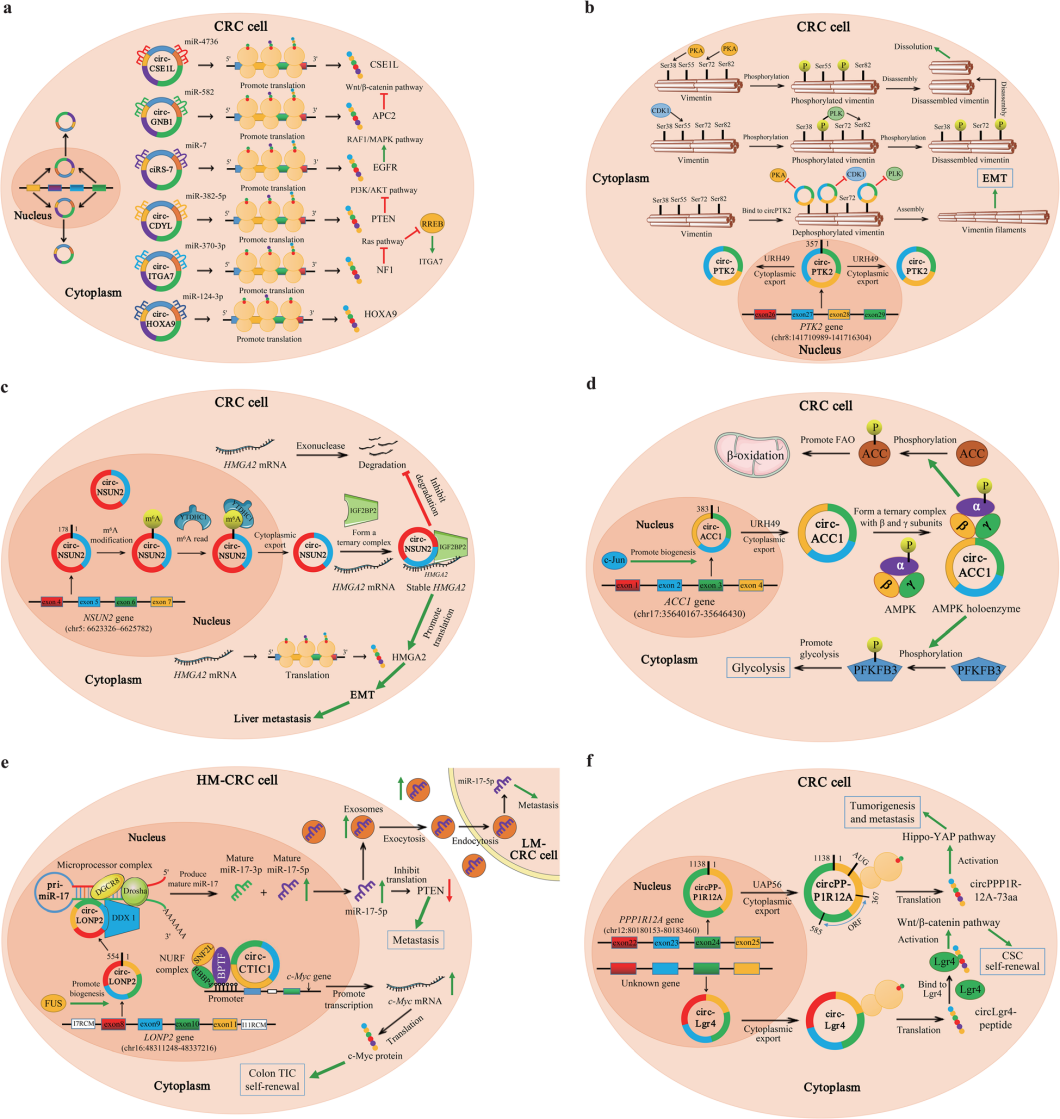
Six general mechanisms underlying the functions of circRNAs in colorectal cancer. **a** | Function as miRNA sponges or decoys. For example, circCSE1L and circHOXA9 protect their homologous mRNAs from miRNA-mediated degradation by inhibiting miRNA activity. A few circRNAs may also indirectly activate or inactivate vital signaling pathways by suppressing miRNAs. **b** | Function as protein sponges or decoys. In the cytoplasm, circPTK2 physically interacts with the vimentin protein at phosphorylation sites Ser38, Ser55 and Ser82, protecting vimentin from phosphorylation by PKA, CDK1 or PLK. **c** | Enhances protein function. As illustrated in the figure, circNSUN2 interacts with IGF2BP2 and *HMGA2* mRNA and forms a circNSUN2/IGF2BP2/*HMGA2* RNA-protein ternary complex, increasing the stability of the *HMGA2* mRNA. **d** | Function as a protein scaffold. In response to energy stress, circACC1 binds the regulatory β and γ subunits of AMPK and promotes the assembly, stabilization, and activity of the AMPK holoenzyme. **e** | Function as protein recruiters. In the nucleus, circLONP2 recruits DGCR8 and the Drosha complex to the primary miR-17 (pri-miR-17) in a DDX1-dependent manner and facilitates the maturation and processing of pri-miR-17. Then, the upregulated mature miR-17-5p not only promotes the metastasis of highly metastatic CRC (HM-CRC) cells by inhibiting PTEN translation but is also assembled into exosomes and internalized by low metastatic CRC (LM-CRC) cells to enhance their aggressiveness. Another example is circCTIC1, which interacts with the NURF complex through BPTF, recruits the NURF complex to the c-Myc promoter and finally drives the transcriptional initiation of the *c-Myc* gene. **f** | Function as translation templates. circPPP1R12A carries a short 216-nt open reading frame (ORF) and encodes a functional protein (named circPPP1R12A-73aa). Another circRNA with peptide-coding capacity is circLgr4

In an authoritative review, Kristensen et al. summarized six general mechanisms of circRNA functions, including acting as (i) miRNA sponges or decoys, (ii) protein sponges or decoys, (iii) protein function enhancers, (iv) protein scaffolds, (v) protein recruiters, and (vi) translation templates [128]. Mechanistic studies published to date reveal that the dysregulated circRNAs in CRC may exert their biological functions through these molecular mechanisms (Fig. 5; Additional file 2).

##### miRNA sponge or decoy

The competitive endogenous RNA (ceRNA) hypothesis posits that transcripts with shared miRNA binding sites compete for posttranscriptional control; specifically, RNAs affect miRNA activity through sequestration, thereby indirectly mediating the posttranscriptional upregulation of miRNA target mRNAs [129]. This hypothesis has attracted notable attention as a unifying function of lncRNAs, pseudogene transcripts and circRNAs, as well as an alternative function of mRNAs [129, 130]. The human genome contains more than 500 miRNA-encoding genes, and miRNAs from individual gene families are able to target and modulate the stability and translation of hundreds of different mRNAs [131, 132]. Many circRNAs contain various types and numbers of miRNA binding sites that specifically bind miRNAs, thereby reducing miRNA activity and upregulating the expression of miRNA target genes. As more than half of all human mRNAs are estimated to be conserved miRNA targets [130], circRNAs are thought to exert widespread effects by regulating gene expression.

In CRC, most circRNAs identified to date have been proposed to function as miRNA sponges or decoys (Fig. 5a; Additional files 2, 4, 5). Some circRNAs even bind several miRNAs, as exemplified by circCCDC66, which contains target sites for miR-33b, miR-93, miR-510-5p and miR-338-3p [112–114]. Thus, these circRNAs may perform various functions by sponging different miRNAs. As shown in a study by Hu et al., hsa_circ_0001461 (circFAT1) upregulates UHRF1 to modulate CRC cell proliferation, apoptosis, and glycolysis by targeting miR-520b and miR-302c-3p [84]. Ren et al. revealed that hsa_circ_0001178 functions as a ceRNA for miR-382/587/616 to upregulate ZEB1 (a key trigger of EMT), subsequently facilitating the metastatic dissemination of CRC [119]. In addition, specific circRNAs promote the transcription of their host genes [89] or protect their homologous mRNAs from miRNA-mediated degradation by inhibiting miRNA activity [105, 133]. For example, Wang et al. revealed that hsa_circ_0060745, a circRNA derived from exons 9 and 10 of CSE1L, functions as a ceRNA to sequester miR-4736 and promotes the CSE1L-mediated proliferation and metastasis of CRC cells [133]. Lai et al. concluded that hsa_circ_0079662 (circHOXA9), a ceRNA that binds hsa-mir-324-5p, upregulates the expression of the target gene HOXA9 and induces oxaliplatin resistance in human colon cancer through the TNF-α pathway [105]. Furthermore, a few circRNAs may indirectly activate or inactivate several vital signaling pathways, such as the Wnt/β-catenin pathway [73, 94, 101, 134–136], EGFR/RAF1/MAPK pathway [46], PTEN/PI3K/AKT pathway [92], Ras pathway [89], etc., by suppressing miRNAs. For instance, Fang et al. revealed that hsa_circ_0013339 (circRNA_100290) functions as a ceRNA for FZD4 by sponging miR-516b, leading to the activation of the Wnt/β-catenin pathway [134]. Li et al. observed the binding of hsa_circ_0026782 (circITGA7) to miR-370-3p to antagonize its suppression of NF1, which is a well-known negative regulator of the Ras pathway; furthermore, circITGA7 upregulates the expression of ITGA7 by suppressing RREB1 through the inhibition of the Ras pathway [89].

##### Protein sponge or decoy

Individual circRNAs containing RBP binding motifs may function as sponges or decoys of these proteins and indirectly regulate RBP-dependent functions (Fig. 5b; Additional file 2). One example is hsa_circ_0005273 (circPTK2), which is backspliced from three exons (exons 27, 28, and 29) of PTK2 [137]. Vimentin is a type 3 intermediate filament protein that is responsible for maintaining the cell shape and stabilizing cytoskeletal interactions. Vimentin is a critical mesenchymal marker in EMT and serves as an organizer of many key proteins involved in cell attachment and migration [138]. The phosphorylation of serine residues in vimentin inhibits subunit polymerization, subsequently promoting the disassembly of vimentin filaments and increasing the solubility of the protein [139]. Yang et al. described the physical interaction between circPTK2 and the vimentin protein at the phosphorylation sites Ser38, Ser55 and Ser82, protecting vimentin from phosphorylation by PKA, CDK1 or PLK; then, vimentin promotes EMT in CRC cells both in vitro and in vivo [137].

##### Enhancer of protein function

As a structural component of protein complexes, circRNAs may interact with and enhance the function of specific proteins (Fig. 5c; Additional file 2). Notably, hsa_circ_0007380 (circNSUN2), also known as circRNA_103783, is located on the chromosome 5p15.31 amplicon of CRC and is derived from exon 4 and 5 regions within the NSUN2 locus [140]. Chen et al. revealed that circNSUN2 was mainly localized in the cytoplasm but was also present in the nucleus [140]. Moreover, YTHDC1, also known as an N^6^-methyladenosine modification (m^6^A) reader, promotes the cytoplasmic export of m^6^A-modified circNSUN2 by interacting with the GAACU m^6^A motif within the exon 5-exon 4 junction site of circNSUN2 [140]. Furthermore, IGF2BP2, an RBP essential for mRNA stability [141, 142], binds the CAUCAU motif of circNSUN2 through the KH3–4 di-domain; then, circNSUN2/IGF2BP2 forms an RNA-protein complex in the cytoplasm [140]. More importantly, the AAACA site of circNSUN2 directly binds the 3′UTR of *HMGA2* with AU-rich elements [140]; HMGA2 has been reported to induce EMT and contribute to colon cancer progression [143]. Based on these results, circNSUN2 plays a critical role in the liver metastasis of CRC by promoting interactions between IGF2BP2 and *HMGA2* and increases the stability of *HMGA2* mRNA through the formation of a ternary circNSUN2/IGF2BP2/*HMGA2* RNA-protein complex.

##### Protein scaffold

Specific circRNAs (e.g., circAmotl1 and circFoxo3) function as protein scaffolds to promote the colocalization of enzymes (such as acetylases, phosphatases and ubiquitin ligases) and their substrates to alter reaction kinetics [144, 145] (Fig. 5d; Additional file 2). Notably, hsa_circ_001391 (circACC1) is generated from exons 2, 3, and 4 of the ACC1 gene, which is located on human chr17 and does not share homology with mouse sequences [52]. AMP-activated protein kinase (AMPK) is among the most well-known metabolic enzymes and plays a crucial role in maintaining energy homeostasis [146]. In response to energy stress, AMPK, which is a critical sensor of the cellular energy status, phosphorylates and activates PFK-2, which stimulates glycolysis [147, 148], while AMPK inhibits fatty acid synthesis (FAS) and stimulates fatty acid oxidation (FAO) by phosphorylating ACC1 and ACC-2, respectively [149]. In a study by Li et al., circACC1 bound the regulatory β and γ subunits and, as a result of this interaction, promoted the assembly, stabilization, and activity of the AMPK holoenzyme in HCT116 cells [80]. Moreover, the unassociated subunits were subjected to ubiquitination and proteasomal degradation, as previously described [150, 151]. Furthermore, the knockdown of circACC1 not only decreased the phosphorylation of ACC1 but also significantly decreased the phosphorylation of PFKFB3 at Ser461 [80], which is one of two PFK2 isoforms regulated by AMPK and a proxy measure of PFK2 activation [147, 148]; in contrast, the overexpression of circACC1 increased PFKFB3 phosphorylation [80]. Collectively, circACC1 not only functions as a scaffold to mediate holoenzyme assembly and stability but also increases AMPK activity, which subsequently promotes glycolysis and FAO in CRC.

##### Protein recruitment site

In addition to their other functions, circRNAs may recruit specific proteins to certain loci or subcellular compartments (Fig. 5e; Additional file 2) as exemplified by *FLI1* exonic circular RNA (FECR1), which recruits methylcytosine dioxygenase TET1 to the promoter region of its own parental gene, resulting in the demethylation of CpG sites and active transcription of the *FLI1* gene [152]. Han et al. identified a high level of hsa_circ_0008558 (circLONP2) expression in metastasis-initiating subgroups of CRC and an essential role in the metastasis of CRC cells [118]. Mechanistically, circLONP2 directly interacts with and promotes the processing of primary miR-17 by recruiting DGCR8 and the Drosha complex in a DDX1-dependent manner [118]. Meanwhile, overexpressed mature miR-17-5p is assembled into exosomes and internalized by neighboring cells to enhance their aggressiveness [118]. In addition, Zhan et al. observed a higher expression of hsa_circRNA_103809, also known as circCTIC1, in colon tumor-initiating cells (TICs), which drove the self-renewal of colon TICs in a c-Myc-dependent manner [86]. Specifically, circCTIC1 interacts with the NURF complex through BPTF (the core component of NURF complex), recruits the NURF complex to the c-Myc promoter and finally drives the transcriptional initiation of c-Myc [86].

##### Translation template

Although most circRNAs are thought to be noncoding because they lack elements essential for cap-dependent translation, such as the 5′ cap and the 3′ poly (A) tail [10, 23, 24, 153, 154], a subset of circRNAs with initial codon sites (AUG), an open reading frame (ORF) and internal ribosome entry site (IRES) elements may be translated under certain circumstances, producing unique peptides with special functions [14, 155–159] (Fig. 5f; Additional file 2). To date, only a few endogenous circRNAs, such as circSHPRH, circFBXW7, circLINC-PINT, circAKT3 and circβ-catenin, have been shown to function as protein templates in human cancers [160], although thousands of circRNAs are predicted to include a putative ORF with an upstream IRES [161]. As circRNA-derived peptides (e.g., FBXW7-185aa) are often shorter than their related linear mRNA-translated proteins and lack essential functional domains (e.g., WD-40 domains), they may function as dominant-deleted protein variants, decoys or regulators of alternative proteins [157]. The first translated circRNA identified in CRC was hsa_circ_0000423 (named circPPP1R12A), and its expression is significantly increased in colon cancer tissues [162]. Zheng et al. identified an ORF in circPPP1R12A, which encoded a functional protein (named circPPP1R12A-73aa) [162]. Moreover, the authors confirmed that circPPP1R12A-73aa, but not circPPP1R12A, promoted the proliferation, migration and invasion of colon cancer by activating the Hippo-YAP signaling pathway [162]. Zhi et al. also identified a novel circRNA, circLgr4 (hsa_circ_02276), with a peptide-coding capacity. Furthermore, circLgr4 was expressed at high levels in colorectal cancer stem cells (CSCs) and drove colorectal CSC self-renewal, tumorigenesis and invasion in a peptide-dependent manner [85]. Importantly, the circLgr4-derived peptide interacted with and activated Lgr4, which further promoted the activation of the Wnt/β-catenin signaling pathway [85].

##### Other/unclear mechanisms

Importantly, circRNAs may regulate the expression of cell cycle-related proteins (e.g., p53 and p16) [72, 125], apoptosis-related proteins (e.g., Bax and Bcl-2) [97, 163] and EMT-related proteins (e.g., MMP2, MMP-9, and E-cadherin) [164–166] to mediate cell cycle arrest, apoptosis, and EMT. Additionally, circRNAs directly regulate CRC-associated signaling pathways, such as the Wnt/β-catenin pathway [76, 78, 167] and PTEN/Akt/mTOR pathway [168, 169]. However, the specific mechanisms underlying these circRNA functions are still unknown.

Notably, hsa_circ_0001946 (ciRS-7), originating from chrX, has been shown to be involved in the growth and metastasis of CRC through various mechanisms. In a previous study, ciRS-7 was significantly upregulated in CRC, and the overexpression of ciRS-7 in CRC cells led to the suppression of miR-7, resulting in a more aggressive oncogenic phenotype [46]. This proposal was supported by two other studies showing that ciRS-7 contributed to the proliferation and metastasis of CRC by targeting miR-7 [170] and miR-135a-5p [171], respectively. In contrast, Tanaka et al. did not observe alterations in cell growth or invasion following ciRS-7 overexpression, whereas the ectopic expression of ciRS-7 led to increased levels of CMTM4 and CMTM6, which were recently identified as crucial regulators of PD-L1 protein expression at the cell surface [172]. Accordingly, the cell surface levels of the PD-L1 protein were increased in ciRS-7-overexpressing CRC cells. However, the effects were not reversed by miR-7 overexpression, indicating that the increase in the cell surface level of PD-L1 in the ciRS-7-overexpressing cells was independent of miR-7 function [172].

### Clinical significance of circRNAs in CRC

Compared with other ncRNAs, such as miRNAs and lncRNAs, circRNAs are more suitable as diagnostic and prognostic biomarkers because of their high stability, long half-life (> 48 h), tissue-specific expression, and unique expression signatures, which are significantly associated with cancer progression and outcomes. Moreover, circRNAs are abundant and widely distributed in various tissues and body fluids, including blood, urine, and saliva, and are even enriched in exosomes; thus, they are promising candidates for noninvasive liquid biopsy indicators. Furthermore, numerous circRNAs have been characterized as functional molecules contributing to cancer initiation and progression, making them potential therapeutic targets. Here, we discuss the clinical implications of circRNAs in CRC.

#### circRNAs as promising diagnostic and prognostic biomarkers

First, circRNAs present distinct expression patterns in CRC tissues and blood from patients with CRC compared with normal controls, and thus, they are considered promising tissue or liquid biopsy biomarkers for the diagnosis of CRC (Additional file 3). For example, Hsiao et al. identified increased circCCDC66 expression in colon cancer, and the area under the receiver operating characteristic curve (AUC) was as high as 0.8843 [112]. The AUC of circCCDC66 suggests that 88% of randomly chosen patients with colon cancer will have higher levels of circCCDC66 than randomly chosen normal subjects, indicating that the expression level of circCCDC66 is a good indicator for the detection of colon cancer [173]. Li et al. documented a significant downregulation of both circITGA7 and its linear host gene ITGA7 in CRC tissues, and the AUC of circITGA7 was 0.8791, with a sensitivity of 0.9275 and a specificity of 0.6667, which was much higher than the value of ITGA7 (AUC = 0.7402) [89]. Xie et al. revealed significantly increased serum exosomal hsa_circ_0101802 (circPNN) levels in patients with CRC compared with healthy control groups [71]. A receiver operating characteristic curve (ROC) analysis indicated that circPNN had significant value in diagnosing CRC, with AUCs of 0.855 and 0.826 in the training and validation sets, respectively [71]. Moreover, the AUC of serum exosomal circPNN for early-stage CRC was 0.854, suggesting that serum exosomal circPNN might be a potential noninvasive biomarker for the early diagnosis of CRC [71]. Zhu et al. identified three circRNAs (hsa_circ_0049487, hsa_circ_0066875, and hsa_circ_0007444) as possible novel biomarkers for the prediction of the transition from colonic adenoma to CRC [174].

Second, circRNAs associated with clinicopathological characteristics, such as lymph metastasis, distal metastasis, and postoperative recurrence, may serve as valuable predictive markers in patients with CRC (Additional file 3). For instance, the circPTK2 levels are increased in both CRC tissues and serum and are positively correlated with lymph node or distal metastases; moreover, an ROC analysis showed that circPTK2 is a marker of CRC in patients with nodal (AUC = 0.7249) or distal (AUC =0.7865) metastases [137]. Xu et al. demonstrated that circRNA_0001178 and circRNA_0000826 were significantly upregulated in CRC tissues from patients with liver metastasis compared to patients without liver metastasis, and both circRNAs have the potential to distinguish liver metastases from CRC; the AUCs were 0.945 for circRNA_0001178 and 0.816 for circRNA_0000826 [57]. Ju et al. generated a four-circRNA-based cirScore, including hsa_circ_0122319, hsa_circ_0087391, hsa_circ_0079480, and hsa_circ_0008039, based on an RNA-seq analysis, quantitative validation and clinical information [56]. After assessment in the training cohort and validation in the internal and external cohorts, the authors confirmed that the four-circRNA-based classifier is a reliable tool for the prediction of postoperative disease recurrence in patients with stage II/III colon cancer (AUC = 0.85) [56].

Finally, circRNAs related to the survival time of patients with CRC may represent attractive prognostic biomarkers (Additional file 3). For example, Jin et al. observed significantly upregulated hsa_circ_0005075 expression in CRC tissues; more importantly, a multivariate analysis revealed that the hsa_circ_0005075 expression level was independently associated with overall survival (OS) (HR = 3.237, 95% CI: 1.479–5.158, *P* = 0.003) and disease-free survival (DFS) (HR = 3.452, 95% CI: 1.638–5.438, *P* = 0.001) [78]. A study by Xiao et al. revealed increased hsa_circ_0022382 (circFADS2) expression in 93.5% (187/200) of patients with CRC [175]. Moreover, a multivariate Cox regression analysis revealed that in addition to distant metastasis and the TNM stage, circFADS2 expression was another independent predictor of the CRC prognosis (HR = 6.228, 95% CI: 1.287–30.131, *P* = 0.023) [175]. Furthermore, the ROC curve analyses indicated that the AUC of circFADS2 expression for the prediction of OS was 0.803, which was less than the TNM stage (0.917) and lymphatic metastasis (0.876) but apparently greater than the invasion depth (0.691) and distant metastasis (0.585) [175]. Similarly, a study by Chen et al. identified circ 001971 expression (HR = 6.456, 95% CI: 2.060–20.232, *P* = 0.001) and TNM stage as independent factors of OS with good prognostic capability (AUC = 0.792) [88].

#### circRNAs as potential therapeutic targets

According to our review, more than 70 upregulated circRNAs are actively involved in the tumorigenesis and progression of CRC, and the silencing of these circRNAs exerts opposite effects in vitro and in vivo (Additional file 2). Thus, these oncogenic circRNAs may serve as potential therapeutic targets, and precise interfering RNAs that are delicately designed to accurately target the unique backspliced junction of oncogenic circRNAs may exert an anti-tumor effect. Accumulating studies using animal models, particularly patient-derived xenograft (PDX) mouse models, have revealed that small interfering RNAs (siRNAs) or short hairpin RNAs (shRNAs) specifically targeting oncogenic circRNAs effectively inhibit CRC growth and metastasis [37, 66, 85, 118, 137, 140, 176, 177]. For example, Yang et al. reported that a tail vein injection of an shRNA specifically targeting circPTK2 reduced tumor metastasis in a CRC-PDX model, suggesting that the oncogenic circPTK2 may serve as a potential therapeutic target for CRC metastasis [137]. Chen et al. substantiated their results using an in vivo metastasis PDX model and showed that tumor metastasis was significantly inhibited after the knockdown of circNSUN2 in PDX cells compared to control cells in either liver or lung metastasis models [140]. Similarly, according to a study by Han et al., an antisense oligonucleotide (ASO) targeting circLONP2 dramatically reduced the penetrance of metastasis to distal organs in vivo, including reductions in both the nodule size and numbers [118]. Interestingly, Wang et al. confirmed that the targeting of ciRS-122 by systemically injecting an exosome-delivered siRNA in a mouse model of oxaliplatin-resistant CRC sensitized CRC to oxaliplatin, suggesting a novel potential approach for the reversion of oxaliplatin resistance in CRC [37]. Additionally, certain drugs or compounds may exhibit anticancer activity through a circRNA-associated axis [83, 124, 162]. For instance, Du et al. confirmed that lidocaine decreases circHOMER1 expression in CRC cells and suppresses cell proliferation and aerobic glycolysis by regulating the circHOMER1/miR-138-5p/HEY1 axis, providing a novel treatment option using lidocaine to prevent the progression of CRC [83]. As shown in a study by Zhen et al., the YAP-specific inhibitor peptide 17 dramatically alleviates the effect of circPPP1R12A-73aa on promoting the growth of colon cancer cells [162].

To the best of our knowledge, at least 20 downregulated circRNAs have been verified to negatively regulate the growth and metastasis of CRC (Additional file 2). The induction of the expression of these tumor suppressor circRNAs in CRC cells or tissues might yield substantial antitumor effects partially due to the high stability and long half-life of circRNAs. Li et al. observed that after carbon ion irradiation, the expression of the circRNA CBL.11 was induced in CRC cells and suppressed cell proliferation by sponging miR-6778-5p [111]. In addition, exogenous circRNAs might be delivered by specific vectors containing DNA cassettes designed for circRNA expression or the transfection of purified in vitro-generated circRNAs [178]. A few studies confirmed the synthesis and cloning of circRNA sequences into special plasmid vectors (e.g., pLCDH-cir; Ribobio, Guangzhou, China) for the production of lentiviruses to stably transfect CRC cell lines and constitutively overexpress the desired circRNAs; then, the exogenous circRNA acted as a tumor suppressor by sponging various miRNAs [73, 89, 94, 179]. For this purpose, the introduction of engineered circRNAs as sponges for specific oncogenic miRNAs into CRC cells or tissues might represent a new and efficient approach for future cancer therapy.

Some circRNAs mentioned above, such as hsa_circ_001680 [102], ciRS-122 [37], circCCDC66 [104, 114], and circRNA CBL.11 [111], are related to chemoradiation resistance in CRC (Additional file 2). Therefore, the detection of the expression of these circRNAs may be important for predicting the sensitivity of patients with CRC to chemoradiotherapy in the clinic. Moreover, therapy targeting these circRNAs may regulate the resistance of patients with CRC to chemoradiotherapy. Finally, interventional methods targeting CDR1-AS, which increases the PD-L1 levels on the surface of CRC cells, may increase the effectiveness of current immunotherapy, namely, PD-1/PD-L1 blocking therapies [172].

## Future perspectives

### Insights and limitations of current research

Although substantial progress has been achieved in identifying and characterizing circRNAs, many crucial unknown factors and limitations of research investigating circRNAs’ biological process still need to be addressed in further studies.

First, how are circRNAs exported from the nucleus to the cytoplasm? Most circRNAs accumulate in the cytoplasm, but how their localization or nuclear export is controlled remains largely unknown. A study by Huang et al. showed that a length-dependent evolutionarily conserved pathway mediated by Hel25E and its homologs (UAP56 and URH49) controls the nuclear export of circRNAs [28]. Additionally, RBP-mediated selective transportation [180] or m^6^A modification [28] has been proposed to facilitate the cytoplasmic export of circRNAs. These discoveries are timely, novel, and pivotal and certainly raise more questions for further studies [181, 182]. For example, how do UAP56 and URH49 define long and short circRNAs? Which factor(s) regulate these pathways? Are there additional mechanisms responsible for the nuclear export of circRNAs? The answers to these questions could also shed light on the biogenesis and functions of circRNAs. Additionally, whether UAP56 targeted small molecule inhibitors [183] or specific inhibitors of nuclear export (SINE) compounds [184] can be used to target tumors that overexpress circRNA remains unknown.

Second, how do circRNAs decay? Although several endonucleases (e.g., RNase P and RNase L) have been proposed to trigger the internal cleavage of circRNAs under certain conditions [185], the detailed turnover process is largely unknown. Future studies should focus on which extracellular or intracellular signals initiate circRNA degradation, which endonucleases or other enzymes account for opening the loop, and how the opened fragments are ultimately degraded. Extracellular vesicles or exosomes might be another possible mechanism by which cells clear redundant circRNAs, but the mechanism guiding circRNA exosome assembly, exocytosis and endocytosis is poorly understood.

Third, why are circRNAs dysregulated in tumors? A balance exists among circRNA generation, localization, and degradation, which is elaborately regulated by cis-elements, trans-acting factors, and the tumor microenvironment. When the balance is disrupted, the expression of circRNAs is altered. However, the specific mechanisms underlying circRNA biogenesis, distribution, and degradation remain elusive.

Forth, how do circRNAs exert their functions in tumors? Dysregulated circRNAs play critical roles in cancer metabolism [186], cancer immune escape [187], the tumor microenvironment [188, 189], and chemoradiotherapy resistance [190], but explorations of their underlying mechanisms have simply scratched the surface. Although the ceRNA hypothesis has been proposed as the most common mechanism by which circRNAs perform their functions, this sponge function of circRNAs has been questioned partially because physiological changes in the expression of most individual transcripts (e.g., lncRNAs and circRNAs) do not compromise miRNA activity [129, 191]. Specifically, few circRNAs harbor as many miRNA binding sites for a single miRNA as ciRS-7 [12, 13] and circZNF91 [23, 192], and the abundance of many circRNAs is far less than that of miRNAs, preventing them from achieving the miRNA sponge effect. Thus, the stoichiometric relationship between the miRNA binding sites of circRNAs and the mRNA target sites of miRNAs must be considered before assigning ceRNA functions to circRNAs [130]. Encoding functional peptides or proteins is another novel and critical mechanism by which circRNAs exert their effects in cancers. Recently, Liu et al. even identified an endogenous rolling-translated protein of circular EGFR RNA (circ-EGFR) that sustains EGFR signaling activation and promotes glioblastoma tumorigenicity [193]. Although thousands of circRNAs are predicted to include a putative ORF with an upstream IRES, only a few circRNAs have been shown to be translated into peptides in human cancers [160, 194]. More efforts should be exerted to predict and identify circRNA encoded peptides, as these peptides are highly cancer specific and could become a new resource for anti-tumor protein drug screening.

Finally, how can circRNAs be utilized in clinical practice? Although researchers have suggested that circRNAs represent promising diagnostic and prognostic biomarkers, particularly noninvasive biomarkers (in blood samples), completely noninvasive samples (stool, urine, saliva, etc.) should be promoted for detection and research. Further studies might focus on screening and validating more circRNA biomarker candidates in a large pool of tumor samples. Moreover, targeting oncogenic circRNAs with ASOs at unique backspliced junctions or promoting the effect of tumor suppressor circRNAs may be promising therapeutic strategies for patients with cancer. Excitingly, researchers have designed and constructed artificial circRNAs using enzymatic ligation in vitro [195–197]. Pioneer studies have shown that these synthetic circRNAs can be stably expressed in cancer cells and efficiently function as miRNA sponges (e.g., miR-21 and miR-93) [195, 196] or protein sponges (e.g., hnRNP L) [197]. In the future, artificial circRNAs could be a potential tool in molecular biology and medicine.

### Innovative approaches for future research

We are pleased to report that research investigating circRNAs is progressing at a steady and fast pace, but limitations and challenges in the methodologies of circRNA studies remain [191, 198]. For example, circRNAs are usually of low abundance, and their sequences often overlap with those of their linear counterparts; thus, more sensitive and specific methods are required for their detection. Moreover, the unique structure of circRNAs is the backspliced junction (BSJ) site, leaving very limited choices for specific primer design, probe design and siRNA design. Furthermore, the CRISPR-Cas9 system may be restricted in knockout studies of circRNAs because attempts to delete the whole sequence could disturb the expression of cognate mRNAs and impact specific loss-of-function studies. These challenges call for more advanced technologies for circRNA detection, interference, and other functional studies. Notably, several promising techniques, such as single-cell RNA-seq [199, 200], digital spatial profiling [201–203], and the Oxford Nanopore MinION technology [204, 205], will be helpful in this endeavor. In the future, we expect these technologies to be adapted to allow spatial and temporal gene expression analyses and the quantification of circRNAs. More importantly, Chen and colleagues reported a study in *Nature Methods* showing that the CRISPR-Cas13 technique can be used to knock down circRNAs without any influence on homologous mRNAs by using guide RNA targeting sequences spanning BSJ sites featured in circRNAs [206]. The CRISPR-RfxCas13d system may serve as a useful tool for the discovery and functional study of circRNAs at both the individual and large-scale level in the near future [207]. Finally, an increasing number of online databases have been developed and improved to provide tremendous valuable information, such as Ularcirc [208], IMS-CDA [209], Lnc2Cancer 3.0 [210], LincSNP 3.0 [211], etc. These online resources will be applied for circRNA identification, characterization and functional analyses and serve as tools for investigating the interaction between circRNAs and other molecules (e.g., miRNAs and RBPs).

Current functional, mechanistic, and clinical insights highlight the important roles of circRNAs in cancer, but we propose that these functions are only the beginning. A better understanding of the mechanisms modulating the circRNA fate, the downstream effectors of circRNA regulatory networks, and the clinical relevance of circRNAs in cancer will increase our knowledge of the roles of circRNAs in cancer biology and the development of circRNA-based diagnosis, prognosis, and therapeutic methods for cancer.

## Conclusions

In summary, CRC is a multistep, multistage and multifactor comprehensive hereditary disease, but the specific pathogenic mechanism has not been completely elucidated. Although significant progress has been achieved in the diagnosis and treatment of CRC in recent decades, the prognosis of patients with advanced CRC, particularly patients with distant metastasis, remains poor. Notably, circRNAs constitute a new class of ncRNAs, and their biological functions include various physiological and pathophysiological processes, which have attracted increasing attention. In particular, circRNAs play crucial roles in the occurrence and development of tumors, rendering them hotspots in the field of cancer research. As described in this review, circRNAs are aberrantly expressed in CRC and are associated with the clinicopathological features and prognosis of patients with CRC. More importantly, individual circRNAs may play pro-cancer, anti-cancer, or chemoradiation sensitivity-regulating roles in the tumorigenesis, metastasis and drug resistance of CRC through various molecular mechanisms. These circRNAs may serve as potential diagnostic indicators, prognostic predictors, or therapeutic targets in the clinical diagnosis and treatment of CRC, thus prolonging the survival time and improving the quality of life of patients with CRC.

## Supplementary Information

**Additional file 1.** Expression profiling of circRNAs in colorectal cancer [212–223].

**Additional file 2.** Biological functions and molecular mechanisms of circRNAs in colorectal cancer [224–261].

**Additional file 3.** Clinical significance of dysregulated circRNAs in colorectal cancer [262–265].

**Additional file 4.** Network of circRNA-miRNA-mRNA interactions in colorectal cancer.

**Additional file 5.** Network of circRNA-miRNA-mRNA interactions in colorectal cancer.

## Data Availability

The datasets supporting the conclusions of this article are included within the article and its additional files.

## Abbreviations

CRC: Colorectal cancer
circRNAs: Circular RNAs
ncRNAs: Noncoding RNAs
PCR: Polymerase chain reaction
RBPs: RNA binding proteins
SD: Splice-donor site
SA: Splice-acceptor site
BP: Branch point
EcircRNAs: Exonic circular RNAs
EIcircRNAs: Exon-intron circular RNAs
CiRNA: Circular intronic RNAs
RNase P: Ribonuclease P
MRP: Multidrug resistance-associated protein 1
m6A: N^6^-methyladenosine
YTHDF2: YTH domain-containing family protein 2
miRNA/miR: microRNA
snRNP: Small nuclear ribonucleoprotein
lncRNAs: Long noncoding RNAs
DECs: Differentially expressed circRNAs
TEM: Transmission electron microscopy
CSCs: Cancer stem cells
TICs: Tumor-initiating cells
EMT: Epithelial-mesenchymal transition
5-FU: 5-fluorouracil
siRNA: Small interfering RNA
shRNAs: Short hairpin RNAs
ASO: Antisense oligonucleotide
RCMs: Reverse complementary sequences
hnRNPs: Heterogeneous ribonucleoproteins
ceRNA: Competitive endogenous RNA
FAS: Fatty acid synthesis
FAO: Fatty acid oxidation
AUG: Initial codon sites
IRES: Internal ribosome entry site
ORF: Open reading frames
ROC: Receiver operating characteristic curve
AUC: Area under the curve
TNM: Tumor-node-metastasis
CEA: Carcinoembryonic antigen
CA19–9: Carbohydrate antigen 19–9
OS: Overall survival
DFS: Disease free survival
HR: Hazard ratio
PDX: Patient-derived xenograft
