# Supplementary figures and images for: Comprehensive landscape and future perspectives of circular RNAs in colorectal cancer
Source: Mol Cancer. 2021 Feb 3;20:26. doi: 10.1186/s12943-021-01318-6 (PMC7856739; doi:10.1186/s12943-021-01318-6)

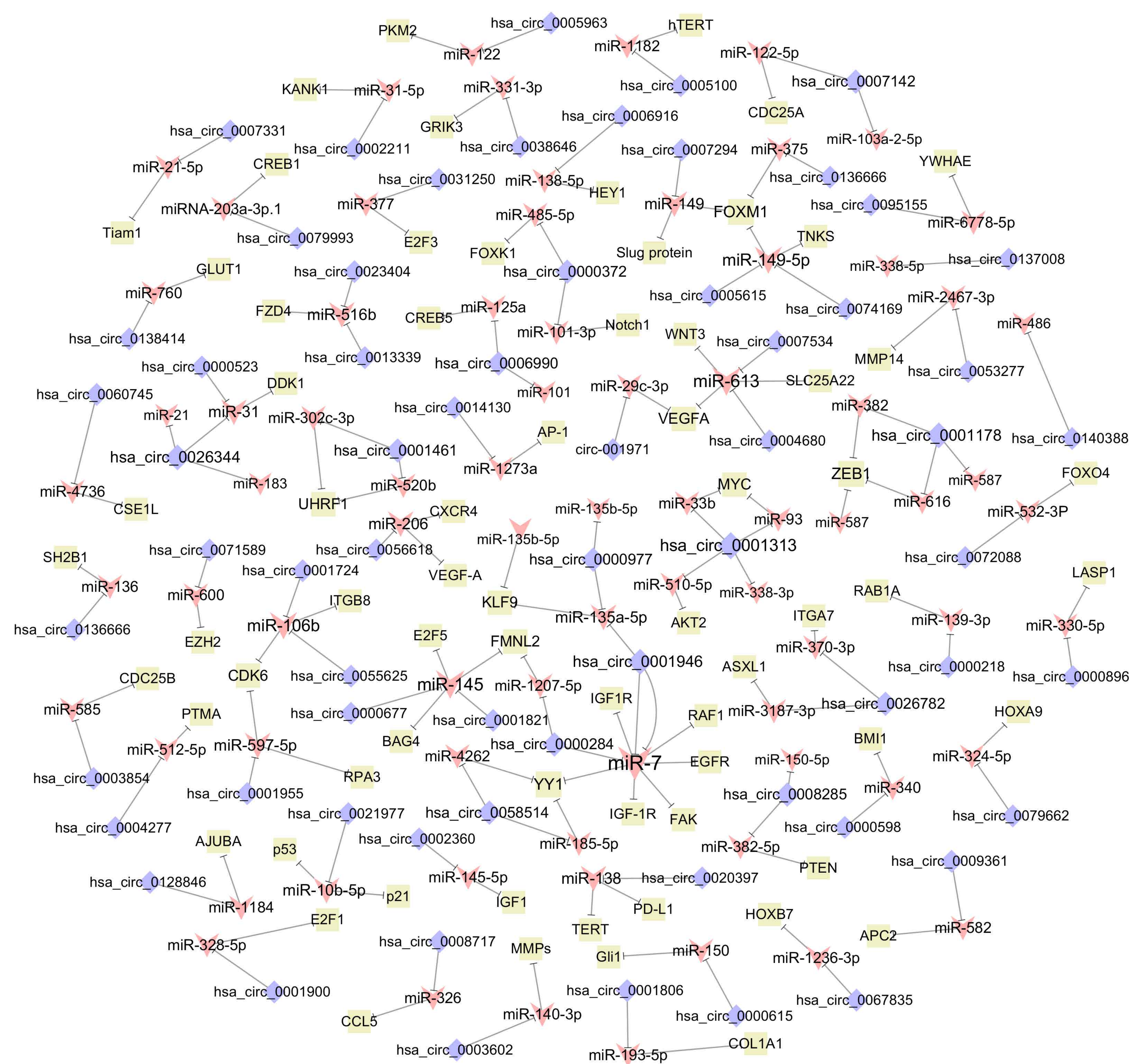

Supplement: Supplementary file 4 — Additional file 4. Network of circRNA-miRNA-mRNA interactions in colorectal cancer. [file 12943_2021_1318_MOESM4_ESM.pdf]
